# Meta-analysis and systematic literature review of COVID-19 associated bradycardia as a predictor of mortality

**DOI:** 10.1186/s43044-022-00284-8

**Published:** 2022-06-04

**Authors:** Chukwuemeka A. Umeh, Sabina Kumar, Elias Wassel, Pranav Barve

**Affiliations:** 1 Department of Internal Medicine, Hemet Global Medical Center, 1117 E. Devonshire Ave., Hemet, CA 92543 USA; 2 St. George’s University, School of Medicine, University Centre Grenada, West Indies, Grenada

**Keywords:** COVID-19, Bradycardia, Bradyarrhythmia, Mortality, Meta-analysis

## Abstract

**Background:**

Cardiac arrhythmias have been identified as independent predictors of mortality in Coronavirus disease 2019 (COVID-19) patients. While some studies have reported poor prognosis with bradycardia in COVID-19 patients, others have not found any association between bradycardia and mortality in COVID-19 patients. This study aims to assess the relationship between bradycardia and mortality in COVID-19 patients by reviewing existing literature.

**Main body:**

Articles were obtained by systematically searching the PubMed and Google scholar databases. Qualitative and quantitative analyses of the studies on bradycardia and mortality in COVID-19 were done. A pooled estimate, with a sample size of 1320 patients, comparing the effect of patients that were bradycardic during their admission with those that were not on mortality showed that bradycardia did not lead to increased mortality in COVID-19 patients (OR 1.25, 95% CI 0.41–3.84, *p* = 0.7).

**Conclusions:**

This meta-analysis showed that bradycardia was not significantly associated with mortality in COVID-19 patients. However, this study is limited by the few studies on bradycardia and mortality in COVID-19 patients. Therefore, future studies should investigate this relationship so that clinicians can prognostically triage and treat COVID-19 patients appropriately.

## Background

Coronavirus disease 2019 (COVID-19), a disease caused by severe acute respiratory syndrome coronavirus 2 (SARS-CoV-2), has affected over 260 million individuals and led to 5.2 million deaths worldwide as of late November 2021 [1]. COVID-19 is primarily considered a respiratory illness; however, it can affect multiple organ systems in the body through a systemic inflammatory response [2]. COVID-19 may result in fever, hypoxia from lung damage from the virus or the host inflammatory response, acute respiratory distress syndrome (ARDS), or multiple organ failure [3]. Additionally, COVID-19 has also been implicated in myocardial injury [4]. The myocardial damage is believed to be caused by direct virus invasion of the myocardium or by the body's inflammatory response to the virus [5]. Consequently, the fever, hypoxemia, and myocardial injury associated with COVID-19 can easily cause arrhythmia [4].

Clinicians worldwide have reported different arrhythmias in COVID-19 patients with atrial arrhythmias, sinus bradycardia, and complete heart block being the most common tachyarrhythmias and bradyarrhythmias reported [6–8]. Similarly, research studies have also reported cardiac arrhythmias, including atrial fibrillation [9, 10], ventricular arrhythmia [3], sinus tachycardia [3], and sinus bradycardia [11], as independent predictors of mortality in COVID-19 patients. While some studies have reported poor prognosis with bradycardia in COVID-19 patients [11, 12], others have not found any association between bradycardia and mortality in COVID-19 patients [10, 13]. This study aims to assess the relationship between bradycardia and mortality in COVID-19 patients by reviewing existing literature.

## Main text

### Study design and outcome

Our meta-analysis was designed according to the guidelines included in the PRISMA statement. Our study hypothesis is that bradycardia in COVID-19 patients is associated with mortality. A study protocol was not registered prior to the study.

### Literature search and data extraction

Articles were obtained on April 10, 2022, by searching PubMed database and Google scholar titles with the keywords: bradycardia and COVID-19, bradycardia and COVID-19 mortality, bradyarrhythmia and COVID-19, bradyarrhythmia and COVID-19 mortality. The articles were manually reviewed by one of the authors and information on the number of patients that reported bradycardia and mortality was extracted into a datasheet in excel (Appendix 1). We excluded studies not written in English.

### Methods for assessing the quality of studies and risk of bias

We assessed the quality of the primary studies using the National Institute of Health Study Quality Assessment Tools [14]. Two authors independently assessed the quality of the primary studies. In case of disagreement between the two authors, the matter was discussed and decided by consensus. The presence of publication bias for each outcome was assessed using funnel plots.

### Data synthesis

We did qualitative data analysis by reviewing the studies, extracting essential information, and describing the similarities and differences in the study outcomes. Additionally, we calculated the odds ratio (OR) and 95% confidence interval (CI) for mortality in patients with and without bradycardia in each study and the combined composite odds ratio for mortality in the studies. We used the random effect model and tested the null hypothesis using Z-test. A *p* value of < 0.05 was interpreted as statistically significant. We tested heterogeneity in study outcomes using the Chi-square test and the *I*^2^ statistic. We did not perform a sensitivity analysis because of the limited number of studies in the analysis. The analysis was done using Comprehensive Meta-Analysis Version 3.

### Identification of relevant studies

**Fig. 1:**
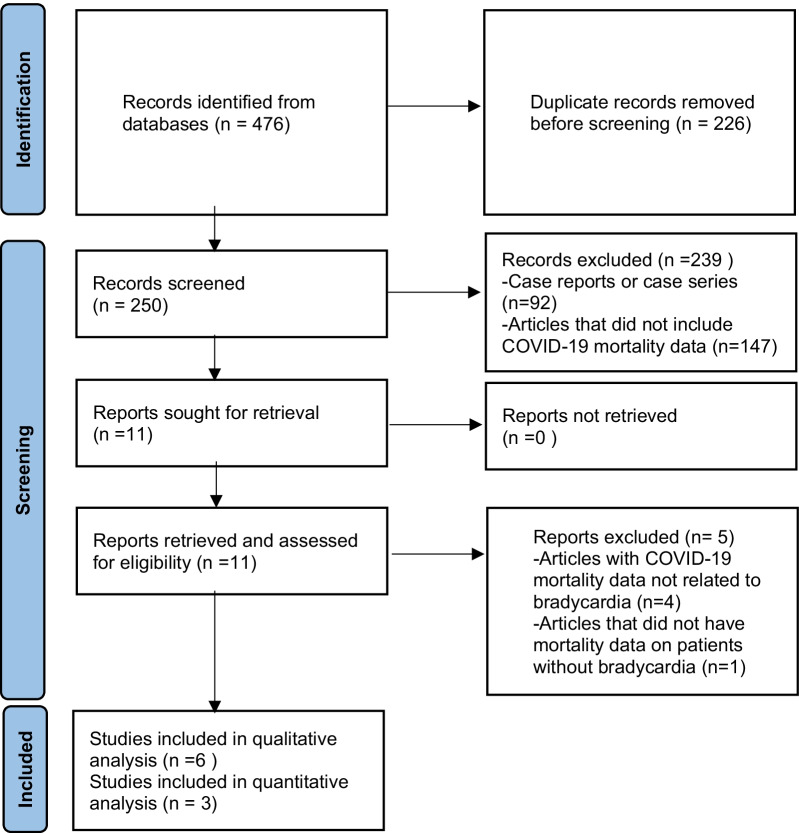
Preferred Reporting Items for Systematic Review and Meta-Analyses guidelines (PRISMA) flowchart of the selection process

Our search produced 476 articles, of which 250 were unique articles after the removal of duplicate publications. We screened the 250 abstracts to assess if they met the inclusion and exclusion criteria in the review, after which we reviewed the full text of 11 articles. Articles excluded include case reports or case series (*n* = 92), articles that did not include COVID-19 mortality data (*n* = 147), articles with COVID-19 mortality data not related to bradycardia (*n* = 4), and articles that did not have mortality data on patients without bradycardia (*n* = 1) (Fig. 1).

In our qualitative analysis, we reviewed six studies on COVID-19 associated with bradycardia and mortality, including two conference abstracts and a study on relative bradycardia. However, our meta-analysis analyzed three studies on bradycardia and mortality in COVID-19 patients, excluding the conference abstracts and the study on relative bradycardia. The authors had access to the full text of all the relevant studies identified by the search strategy. We also made multiple unsuccessful attempts to get more data from the authors of the conference abstracts.

### Risk of bias assessment

**Fig. 2:**
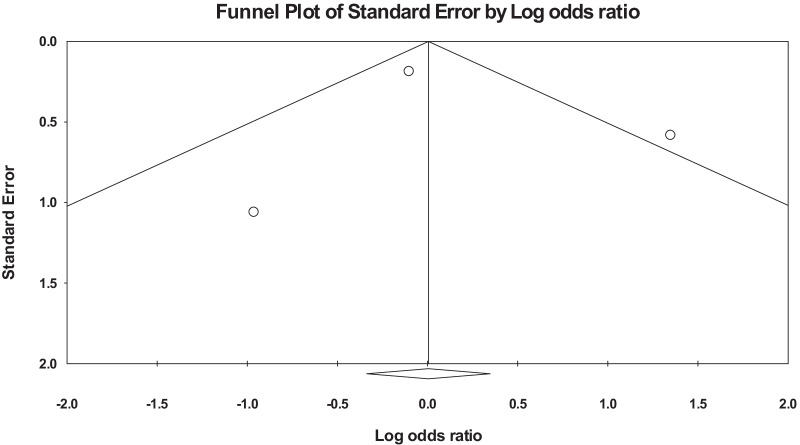
Funnel plot of the primary studies in the meta-analysis

Table 1 shows the quality of each of the three primary studies in the meta-analysis using the National Institute of Health Study Quality Assessment (NIHSQA) Tool for observational cohort and cross-sectional studies. The primary studies in the meta-analysis were of good quality based on the NIHSQA tool for observational cohort and cross-sectional studies with a low risk of within-study bias. The heterogeneity between the studies was considerable with Tau^2^: 0.65; Chi^2^: 6.5; *df*: 2 (*p* = 0.038); and *I*^2^: 69.2, hence the use of the random effect model in our analysis. The funnel plot did not show any significant publication bias in the primary outcome assessed, given the meta-analysis's small number of primary studies (Fig. 2).

**Table 1:** Shows the assessment of the quality of studies in the meta-analysis

Criteria	Study		
	Chalkias et al. [16]	Kumar et al. [11]	Antwi-Amoabeng et al. [10]
1. Was the research question or objective in this paper clearly stated?	Yes	Yes	Yes
2. Was the study population clearly specified and defined?	Yes	Yes	Yes
3. Was the participation rate of eligible persons at least 50%?	Yes	Yes	Yes
4. Were all the subjects selected or recruited from the same or similar populations (including the same time period)? Were inclusion and exclusion criteria for being in the study prespecified and applied uniformly to all participants?	Yes	Yes	Yes
5. Was a sample size justification, power description, or variance and effect estimates provided?	NA	NA	NA
6. For the analyses in this paper, were the exposure(s) of interest measured prior to the outcome(s) being measured?	Yes	Yes	Yes
7. Was the timeframe sufficient so that one could reasonably expect to see an association between exposure and outcome if it existed?	Yes	Yes	Yes
8. For exposures that can vary in amount or level, did the study examine different levels of the exposure as related to the outcome (e.g., categories of exposure, or exposure measured as continuous variable)?	NA	NA	NA
9. Were the exposure measures (independent variables) clearly defined, valid, reliable, and implemented consistently across all study participants?	Yes	Yes	Yes
10. Was the exposure(s) assessed more than once over time?	NA	NA	NA
11. Were the outcome measures (dependent variables) clearly defined, valid, reliable, and implemented consistently across all study participants?	Yes	Yes	Yes
12. Were the outcome assessors blinded to the exposure status of participants?	NR	No	NR
13. Was loss to follow-up after baseline 20% or less?	Yes	Yes	Yes
14. Were key potential confounding variables measured and adjusted statistically for their impact on the relationship between exposure(s) and outcome(s)?	Yes	Yes	Yes

^*^NA, not applicable; NR, not reported

## Qualitative result

### Bradycardia and mortality in COVID-19 patients

We found three studies and two conference abstracts on bradycardia and mortality in COVID-19 patients. The two conference abstracts, Zaidi et al. and Farouji et al., and one of the observational studies, Antwi-Amoabeng et al., found no statistically significant association between bradycardia and mortality [10, 13, 15]. However, the large multicenter study by Kumar et al. found a significant association between bradycardia and mortality when bradycardic patients were compared with patients with normal heart rates [11]. Similarly, Chalkias et al. found that the onset of sinus bradycardia during their COVID-19 admission was associated with mortality in intensive care patients. [16]

### Brief description of studies on bradycardia and mortality in COVID-19 patients

Kumar et al. studied bradycardia as a predictor of mortality in COVID-19 patients. The study was a multi-site retrospective observational study that included seven hospitals in Southern California and involved 1053 patients. Bradycardia was defined as a heart rate less than 60 beats per minute during continuous heart monitoring. Twenty-five percent of patients had bradycardia. The study found that patients with bradycardia were 6.59 times (95% CI [2.83–15.36]) more likely to die than a sub-set of patients with normal heart rates. Their study also showed no statistically significant correlation between hypoxia and bradycardia. However, the study did not report the odds of death of bradycardic patients compared to all those without bradycardia, including those with normal heart rate and those with tachycardia. [11]

Chalkias et al. studied the incidence of sinus bradycardia and its effect on mortality. The study was a single-center study involving 81 patients that required ICU level of care. After adjusting for different confounding factors, they found that the onset of sinus bradycardia was associated with mortality (*p* < 0.001). However, all the patients in the study eventually had bradycardia. Bradycardia was determined by a heart rate of less than 60 during continuous heart monitoring. They also found that sinus bradycardia was not associated with myocardial necrosis or the use of medication that induces bradycardia. [16]

Antwi-Amoabeng et al. studied the association between electrocardiographic features and mortality in 186 COVID-19 patients. An electrocardiogram (ECG) was done on the patients after testing positive for COVID-19. Only seven percent of the patients had sinus bradycardia on ECG. Bradycardia was not associated with mortality. However, A-Fib, atrial flutter, and ST-segment depression were found to be predictive of mortality. [10]

Zaidi et al. studied the association between sinus bradycardia and the severity of COVID-19 infection, including survival outcomes. The study was a multi-site retrospective observational study that included four hospitals and involved 1415 patients. They found that patients with incident bradycardia on three consecutive days were more likely to require ICU admission than those without these events (*p* = 0.001). However, there was no significant association between bradycardia and survival to hospital discharge (*p* = 0.761). Conversely, they found that tachycardia on day-3 of admission compared to normal heart rate was statistically significantly associated with 7-day mortality (*p* < 0.001).

Farouji et al. studied the relationship between transient sinus bradycardia on initial admission and clinical outcomes in 490 COVID-19 patients. They found that patients with transient sinus bradycardia on hospital admission were more likely to require intubation than those without it (*p* = 0.041). Additionally, transient sinus bradycardia on admission was associated with the risk of mortality (*p* = 0.052), though not statistically significant.

### Relative bradycardia and mortality in COVID-19 patients

Oliva et al. studied the association between relative bradycardia and mortality in 101 COVID-19 patients with a fever of ≥ 38.3 °C. In the study, relative bradycardia was defined as heart rate < 90 bpm and concomitant fever (temperature ≥ 38.3 °C). Forty-two percent of these patients had relative bradycardia. They found that ICU admission and in-hospital mortality were not associated with relative bradycardia. [17]

### Quantitative result

**Fig. 3:**
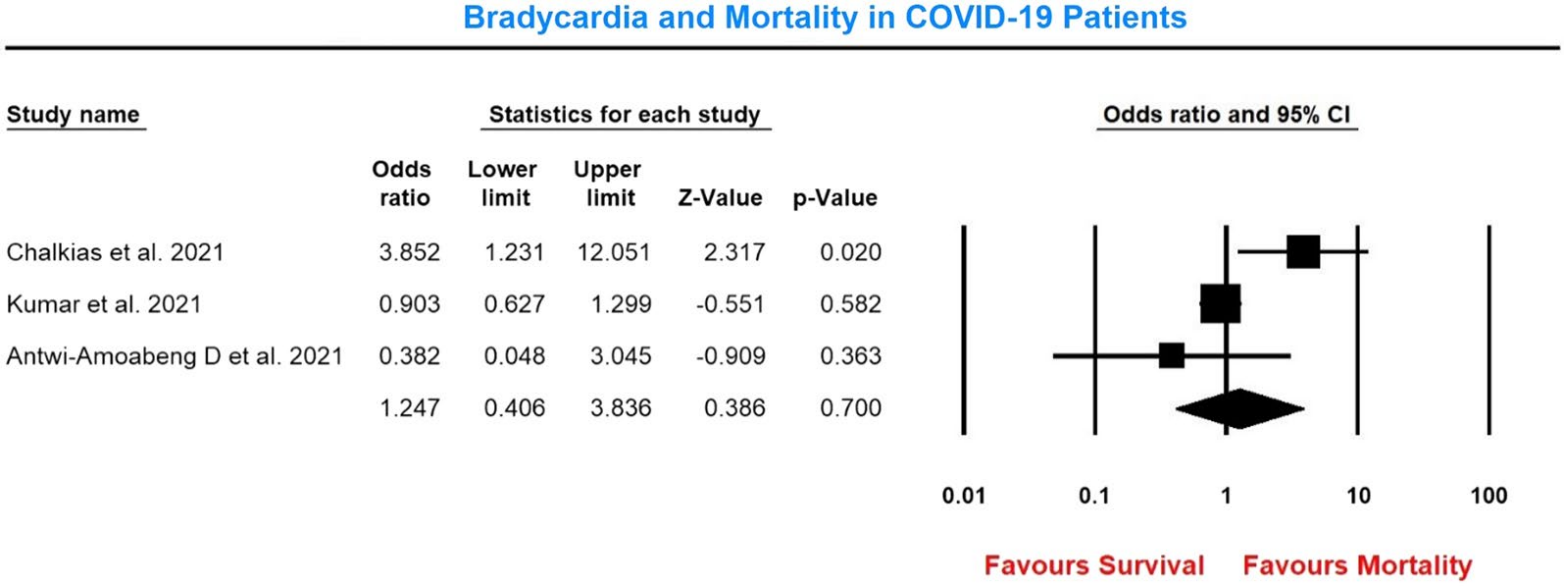
Forest plot showing bradycardia and mortality in COVID-19 patients. Bradycardia group n/N: 99/339; Non-Bradycardia group n/N: 191/981. Heterogeneity: Tau^2^: 0.65; Chi^2^: 6.5; *df*: 2 (*p* = 0.038); *I*^2^: 69.2

The pooled estimate of the effect of bradycardia on mortality in COVID-19 patients compared to patients who were not bradycardic with 1320 patients showed that bradycardia did not lead to increased mortality in COVID-19 patients (OR 1.25, 95% CI 0.41–3.84, p = 0.7) (Fig. 3).

## Discussion

Our study found that bradycardia was not associated with mortality in COVID-19 patients. This result is contrary to the largest study on bradycardia and mortality by Kumar et al. that showed that bradycardia was predictive of mortality in COVID-19 patients. [11] The possible explanation for this is that Kumar et al. reported bradycardia being associated with mortality when bradycardic patients are compared to a subset of patients with normal heart rates. However, they excluded patients with tachycardia. Our study compared bradycardic patients with non-bradycardic patients, including patients with normal heart rates and tachycardic patients. Tachycardia has been associated with increased mortality in COVID-19 patients [3], and this might have explained why bradycardia was not associated with mortality in our study. Similarly, Olivia A et al. did not find any significant association between relative bradycardia, which is defined as heart rate < 90 bpm, and concomitant fever (temperature ≥ 38.3 °C) and mortality in COVID-19 patients. [17]

The average incidence of bradycardia in the three studies in our meta-analysis was 26%. The large variation in the incidence of bradycardia in the primary studies may be due to the difference in the study design. Kumar et al. and Chalkias et al. used continuous cardiac monitoring to determine bradycardia in all hospitalized COVID-19 patients and patients admitted to the ICU and reported a bradycardia incidence of 24.9% and 79%, respectively. [10, 16] Conversely, Antwi-Amoabeng et al. used ECG done after COVID-19 diagnosis to determine bradycardic patients and reported an incidence of seven percent [10]. Thus, using an ECG done after the COVID-19 diagnosis will likely underestimate the incidence of sinus bradycardia in COVID-19 patients. Conversely, Chalkias et al. measured bradycardia in only ICU patients and will likely lead to over-estimation of bradycardia. [16]

The mechanism of bradycardia in COVID-19 appears to be multifactorial. It could be caused by the direct pathogenic effect of the virus on the myocardium, hypoxia, drug toxicity, severe downregulation of myocardial angiotensin-converting enzyme 2 (ACE2) pathways resulting in myocardial inflammation, or the effect of the inflammatory cytokines on the heart [10, 18, 19]. However, the direct pathogenic effect of the COVID-19 virus on pacemaker cells and the effect of the systemic inflammation on cardiac pacemaker cells are the two most commonly proposed mechanisms of bradycardia [18, 19]. Beta-blockers and hypoxia were not associated with bradycardia in both Kumar et al. and Chalkias et al. studies. [11, 16]

Similarly, relative bradycardia has been reported in COVID-19 patients [17, 20, 21]. Physiologically, the heart rate is expected to increase by about ten beats for each degree increase in temperature in degrees Fahrenheit. Thus, a temperature of 101 °F (38.3 °C) is expected to have a corresponding heart rate of about 110 [22]. Relative bradycardia has been reported in infectious processes like typhoid and Q-fever and non-infectious processes like drug fever. The most common cause of relative bradycardia in patients with fever is the use of beta-blocker medications [22]. However, beta-blockers have not been associated with bradycardia in COVID-19 and are unlikely to cause relative bradycardia seen in COVID-19 patients [11, 16]. The direct pathogenic effect of the COVID-19 virus on cardiac pacemaker cells and/or the effect of inflammatory cytokines on cardiac pacemaker cells has been proposed as possible causes of relative bradycardia. [20, 23]

### Limitations of the study

One of the major limitations is the few studies combined in the meta-analysis due to the limited studies that reported bradycardia and its association with mortality in COVID-19 patients. Thus, the small number of studies and participants may make it difficult to detect an effect, even if one exists, so the results of this analysis should be interpreted with caution. Furthermore, two conference abstracts identified during the literature search were not included in the meta-analysis because of incomplete data, which could potentially bias our study's result. However, both conference abstracts did not find any statistically significant relationship between bradycardia and COVID-19 mortality, which is consistent with the findings of our meta-analysis. Additionally, the heterogeneity of the studies in the meta-analysis is considerable and could have affected the conclusion of our study. However, we mitigated the effect of the heterogeneity in our analysis by using a random-effects model for the meta-analysis, which assumes that the primary studies are heterogeneous and gives a more conservative estimate of effect. Finally, our study is a combination of observational studies, and there could have been unmeasured or unadjusted confounders that affected the study outcome in the primary studies.

### Implications of the results for practice, policy, and future research

Our study showed that bradycardia was not significantly associated with mortality in COVID-19 patients. The implication for practice is that there might be no mortality benefit in aggressively treating asymptomatic sinus bradycardia in COVID-19 patients.

The opportunities for future research include more studies to investigate the relationship between bradycardia and mortality in COVID-19 patients. Some studies have suggested that bradycardia in COVID-19 patients might be harbingers of a more severe disease. Therefore, understanding that association may help clinicians prognostically triage the patients appropriately. Additionally, it may be interesting to see the effect of bradycardia in different sub-groups of COVID-19 patients, such as symptomatic vs. asymptomatic patients and younger vs. older patients.

## Conclusions

The result of this meta-analysis showed that bradycardia was not significantly associated with mortality in COVID-19 patients. However, this study is limited by the few studies on bradycardia and mortality in COVID-19 patients. Therefore, future studies should investigate this relationship so clinicians can prognostically triage and treat COVID-19 patients appropriately.

## Data Availability

Not applicable.

## Appendix

See Table

**Table 2:** Summary of studies included in the meta-analysis

Study	Country of study	Definition of bradycardia	Patients analyzed in the study	Patients that had bradycardia	Patients without bradycardia	Number that died in bradycardia group	Number that died in non-bradycardia group
Chalkias et al. [16]	Greece	Heart rate ≤ 60 BPM on continuous heart rate monitoring	Consecutive patients requiring ICU admission	64	17	52	9
Kumar et al. [11]	USA	Heart rate < 60 BPM on two separate occasions, a minimum of 4 h apart, on continuous cardiac monitoring	All consecutive COVID-19 patients admitted during the study period	262	791	46	151
Antwi-Amoabeng et al. [10]	USA	Bradycardia on cardiologist-confirmed ECG report	All consecutive COVID-19 patients admitted during the study period	13	173	1	31

BPM: beats per minute, ICU: intensive care unit, ECG: electrocardiogram

2.

## Abbreviations

ARDS: Acute respiratory distress syndrome
ACE2: Angiotensin-converting enzyme 2
ARDS: Acute respiratory distress syndrome
CI: Confidence interval
COVID-19: Coronavirus disease 2019
NIHSQA: National Institute of Health Study Quality Assessment
OR: Odds ratio
PRISMA: Preferred reporting items for systematic reviews and meta-analyses
SARS-CoV-2: Severe acute respiratory syndrome coronavirus 2
